# Cardiac sensing at a spinal cord stimulation lead: a promising on-device potential biomarker for pain and wellbeing

**DOI:** 10.3389/fphys.2024.1342983

**Published:** 2024-08-12

**Authors:** AnneMarie K. Brinda, Lisa Goudman, Maarten Moens, Juan Hincapie, David A. Dinsmoor, Leonid M. Litvak, Małgorzata Straka

**Affiliations:** ^1^ Medtronic, Minneapolis, MN, United States; ^2^ STIMULUS Research Group, Vrije Universiteit Brussel, Brussels, Belgium; ^3^ Department of Neurosurgery, Universitair Ziekenhuis Brussel, Brussels, Belgium; ^4^ Cluster Neurosciences, Center for Neurosciences (C4N), Vrije Universiteit Brussel, Brussels, Belgium; ^5^ Pain in Motion (PAIN) Research Group, Department of Physiotherapy, Human Physiology and Anatomy, Faculty of Physical Education and Physiotherapy, Vrije Universiteit Brussel, Brussels, Belgium; ^6^ Research Foundation—Flanders (FWO), Brussels, Belgium; ^7^ Department of Radiology, Universitair Ziekenhuis Brussel, Brussels, Belgium

**Keywords:** spinal cord stimulation, objective measures, heart rate variability, heart rate, wellbeing

## Abstract

**Introduction:** In the search for objective measures of therapeutic outcomes for patients with spinal cord stimulation (SCS) devices, various metrics of cardiac performance have been linked to pain as well as overall health. To track such measures at home, recent studies have incorporated wearables to monitor cardiac activity over months or years. The drawbacks to wearables, such as patient compliance, would be obviated by on-device sensing that incorporates the SCS lead. This study sought to evaluate the feasibility of using SCS leads to record cardiac electrograms.

**Methods:** The quality of signals sensed by externalized, percutaneous leads in the thoracic spine of 10 subjects at the end of their SCS trial were characterized across various electrode configurations and postures by detecting R-peaks and calculating signal-to-noise ratio (SNR). In a subset of 5 subjects, cardiac metrics were then compared to those measured simultaneously with a wearable.

**Results:** The average signal quality was acceptable for R-peak detection (i.e., SNR > 5) for all configurations and positions across all 10 subjects, with higher signal quality achieved when recording in resting positions. Notably, the spinal lead recordings enabled more reliable beat detection compared to the wearable (*n* = 29 recording pairs; *p* < 0.001). When excluding wearable recordings with over 35% missed beats, the inter-beat intervals across devices were highly correlated (*n* = 22 recording pairs; Pearson correlation: R = 0.99, *p* < 0.001). Further comparisons in these aligned wearable and corresponding spinal-lead recordings revealed significant differences in the frequency domain metrics (i.e., absolute and normalized high and low frequency HRV power, *p* < 0.05), but not in time domain HRV parameters.

**Discussion:** The ability of an implanted SCS system to record electrocardiograms, as demonstrated here, could provide the basis of automated SCS therapy by tracking potential biomarkers of the patient’s overall health state without the need for additional external devices.

## Introduction

Spinal Cord Stimulation (SCS) has been used as an effective therapy for chronic pain for over 50 years (Shealy et al., 1967; Sdrulla et al., 2018; Vallejo et al., 2020). The current standards for assessing the effectiveness of pain therapies such as SCS are patient-determined scores such as the Visual-Analog-Scoring (VAS) or Numerical Rating Scale (NRS). These subjective measures do not encompass the broad effects that pain has on overall wellbeing (Haefeli and Elfering, 2006). Accordingly, questionnaires such as the Oswestry Disability Index (ODI) or Patient Reported Outcomes Measurement Information System (PROMIS-29) (Fairbank and Pynsent, 2000; Licciardone et al., 2017; Pope et al., 2021) are used to understand the multidimensional influence of pain and therapy on the patient’s activity, sleep, mood, and social engagement (Russo et al., 2020; Pilitsis et al., 2021; Goudman et al., 2022; Levy et al., 2023). While they provide more holistic measures of wellbeing, these questionnaires are self-reported and lengthy. The applications of these surveys are best suited towards occasional, interval assessments—rather than continuous and repeated assessments—of healthcare interventions and changes in health (Hays et al., 2018).

Automated, objective feedback of the patient’s response to therapy could further improve outcomes with SCS. To this end, recent research has assessed an assortment of biosignals that may correlate with chronic pain (Eldabe et al., 2022). One such example in chronic pain patients is the overall decrease in parasympathetic-related heart rate variability (HRV) metrics–as assessed with the root mean square of successive differences (RMSSD) and high frequency power of HRV (Hallman et al., 2015; Koenig et al., 2016; Tracy et al., 2016). A potential reversal of this effect has been noted in patients receiving SCS for both chronic pain (Goudman et al., 2019) and refractory angina (Anselmino et al., 2009). These studies have used electrocardiograms (ECG), Holter monitors, or other wearables to collect HRV metrics. While ECG and Holter monitors provide rich, highly accurate data, they are typically limited in study duration; ECG is commonly only measured in-clinic, and Holter monitors are worn over a limited interval (typically 24–48 h but can be up to several weeks) out-of-clinic. Cardiac metrics monitored over months to years—beyond the scope afforded by an ECG or Holter monitor—in the patient’s home environment may give important insights about a patient’s overall wellbeing and response to pain therapies, as well as better align with the individual fluctuations in pain intensity.

To track long-term chronic pain outcomes via potential objective measures, recent studies have leveraged the use of wearables for at-home collection for patients implanted with an SCS device (Reinen et al., 2022; Patterson et al., 2023). However, there are limitations with the analysis used in wearables including the manufacturer-specific calculations and timing of measurements, reliability of heart beat detection, and patient compliance in wearing the device (Han et al., 2022). Thus, for patients implanted with an SCS device, using on-device sensors may improve the ability to track these objective measures of pain. In addition to potentially enabling physicians to better follow long-term outcomes, the signals may also be leveraged in automated remote programming of therapy adjustments.

Just as implantable cardiac pacemakers have incorporated biopotential sensing to assist in therapy optimization (Escher, 1973), some emerging SCS systems can directly sense biopotentials. These systems record the spinal evoked compound action potential (ECAP), a quantitative measure of neural activation elicited by a stimulation pulse (Russo et al., 2018; Vallejo et al., 2021). Interestingly, other biopotentials beyond the spinal ECAP can be sensed with epidurally placed leads. Leads placed in the thoracic spine are well-positioned to detect cardiac signals due to the proximity of the heart. An early feasibility study in pigs has demonstrated that cardiac signals can be detected by SCS leads (Verma et al., 2023). However, to the best of our knowledge, the ability to sense these signals has thus far not been explored in the human chronic pain population.

In this study, we assess the clinical feasibility of acquiring cardiac signals from SCS leads over an assortment of electrode configurations and postures. Additionally, we compare number of beats detected and inter-beat intervals (IBIs) recorded from a wearable to those recorded from the SCS lead. Finally we compare HRV metrics across the time and frequency domain to evaluate whether any potential differences may impact these summary measures.

## Methods

### Study protocol

This study was a Nonsignificant Risk (NSR) device early clinical research feasibility study in which evoked (e.g., ECAPs) waveforms collected with externalized leads were recorded from subjects with chronic pain undergoing a commercial SCS trial according to approved labelling. This multi-center study conducted in the United states was registered with clinicaltrials.gov (NTC 06499220) and was approved by the WIRB Copernicus Group (WCG study#20192352). All research was conducted in accordance with the Declaration of Helsinki. Subjects provided written informed consent prior to participation in study activities and were compensated for their time.

During the study visit, biopotentials from the SCS leads of 10 subjects were collected during various electrode configurations and subject body postures to determine the effect on signal-to-noise ratio (SNR). In a subset of 5 subjects, a comparison was made between IBIs and derived cardiac metrics from SCS leads to those recorded simultaneously using an Apple Watch.

### Research system

Cardiac signals were recorded with a custom research system, as previously detailed in Chakravarthy et al. (2020). Briefly, the cardiac signals were amplified (Digitimer D440) and recorded with a sampling rate of 40 kHz before digitization (Biopac MP160) and storage on a laptop (via Biopac AcqKnowledge software) for further processing. The system also included stimulation components including an isolated, clinical-grade stimulator (Digitimer DS5) and National Instruments hardware. The system was configured to interface with standard, commercially available, 8-electrode, 60 cm-long percutaneous SCS leads (Model 977D260, Medtronic plc) through a Multi-Lead Trialing Cable (MLTC model #3555-31, Medtronic) and custom adapter. Each electrode was 3 mm long with an inter-contact spacing of 4 mm. For monopolar recordings, the amplifier was referenced to a disposable ground patch electrode (40 × 50 mm contact area ground plate electrode with 2.0 m lead length, Natus Neurology Inc.) placed on the subject’s lower back. Signals were recorded in the absence of stimulation except where noted, where 50 Hz, 200 µs stimuli were applied at amplitudes up to 5.5 mA. Stimulation was applied neighboring bipoles at the opposite end of the lead as the recording contacts in either neighboring or half-lead configurations.

### Clinical data acquisition

**FIGURE 1:**
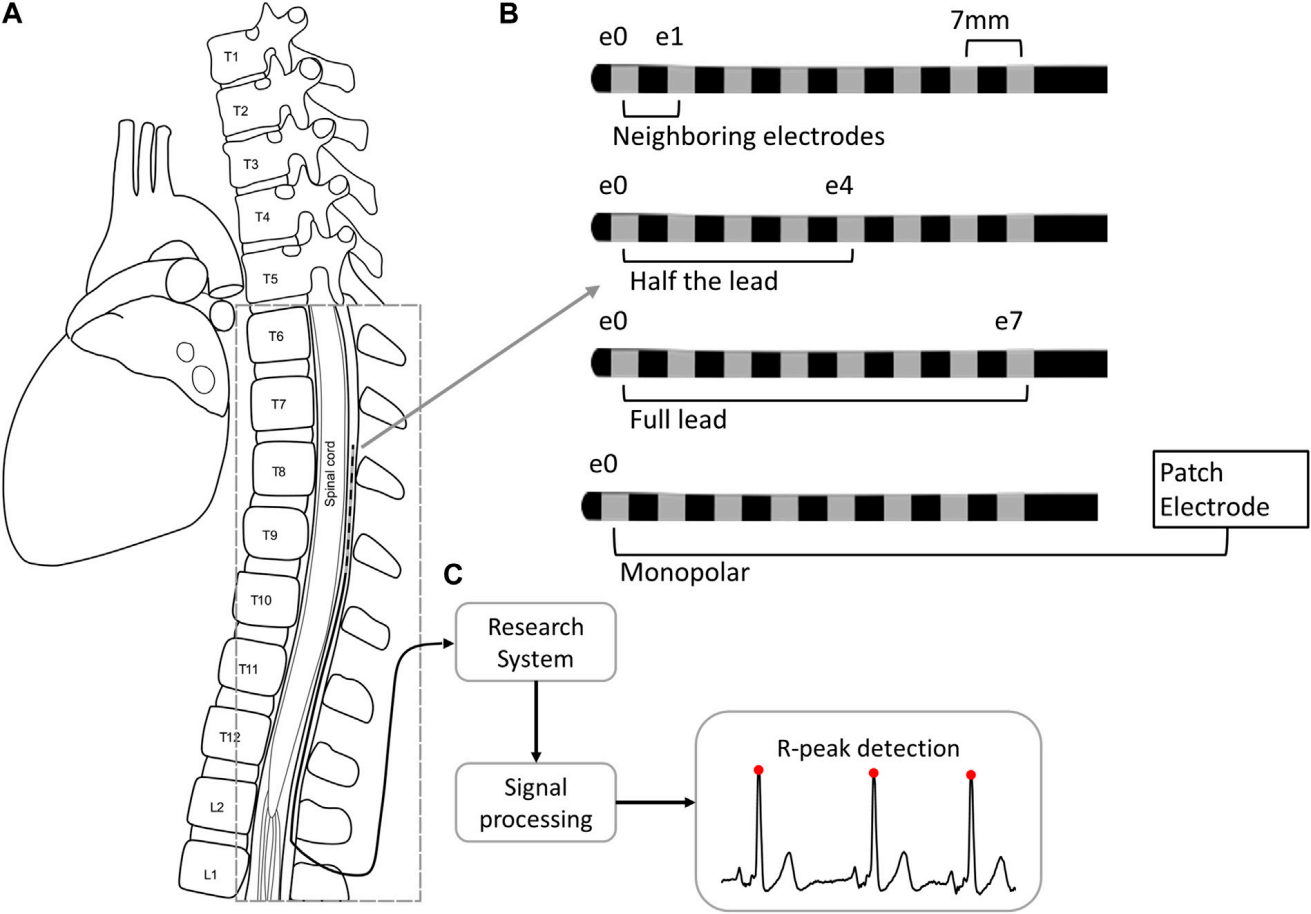
Cardiac signal collection and processing from the SCS lead implanted in the epidural space of the thoracic spine **(A)**. The SCS trial lead has eight 3 mm electrodes with 4 mm spacing. The signals collected from the lead were collected across four different configurations **(B)** and were processed **(C)** to detect R-peaks (red circles). Abbreviations. E: electrode; L: lumbar; T: thoracic.

Data from 10 subjects, all of which had intractable back and/or limb pain, were collected at the end of their SCS trial period prior to the lead pull. Two 8-electrode SCS leads were already implanted in the dorsal epidural space (Figure 1). Images of lead locations taken at the beginning of the study (i.e., at the end of the commercial trial) showed the location ranged from T5 to T11, with 90% of leads being within T7-T11 and the typical lead implanted from T8 to T10. At the end of the SCS trial period and before trial lead removal, the research system was connected to each lead and recordings were collected for at least 30 s.

Given that body position can impact the proximity of the SCS lead to the spinal cord (Cameron and Alo, 1998; Olin et al., 1998; Kuechmann et al., 2009; Abejón et al., 2014), the impact of posture and activity on the fidelity of recordings were evaluated. Specifically, the positions tested included: seated, supine, and walking in place.

Signals were recorded in four different electrode configurations: across the full lead (e0–e7), across half the lead (e0–e4 or e4–e7), with neighboring electrodes (e0–e1 or e6–e7), and monopolar (e.g., e0–patch electrode), where e0 is the cranial-most electrode. Unless otherwise noted, comparisons of the signal quality between configurations was performed across resting positions including seated, recumbent, supine, and prone.

In a subset of subjects (n = 5), spinal lead recordings were done simultaneously with wearable cardiac recordings to compare and validate cardiac metrics. The wearable recording acquisition is detailed further below.

### Analysis of spinal lead recordings

**FIGURE 2:**
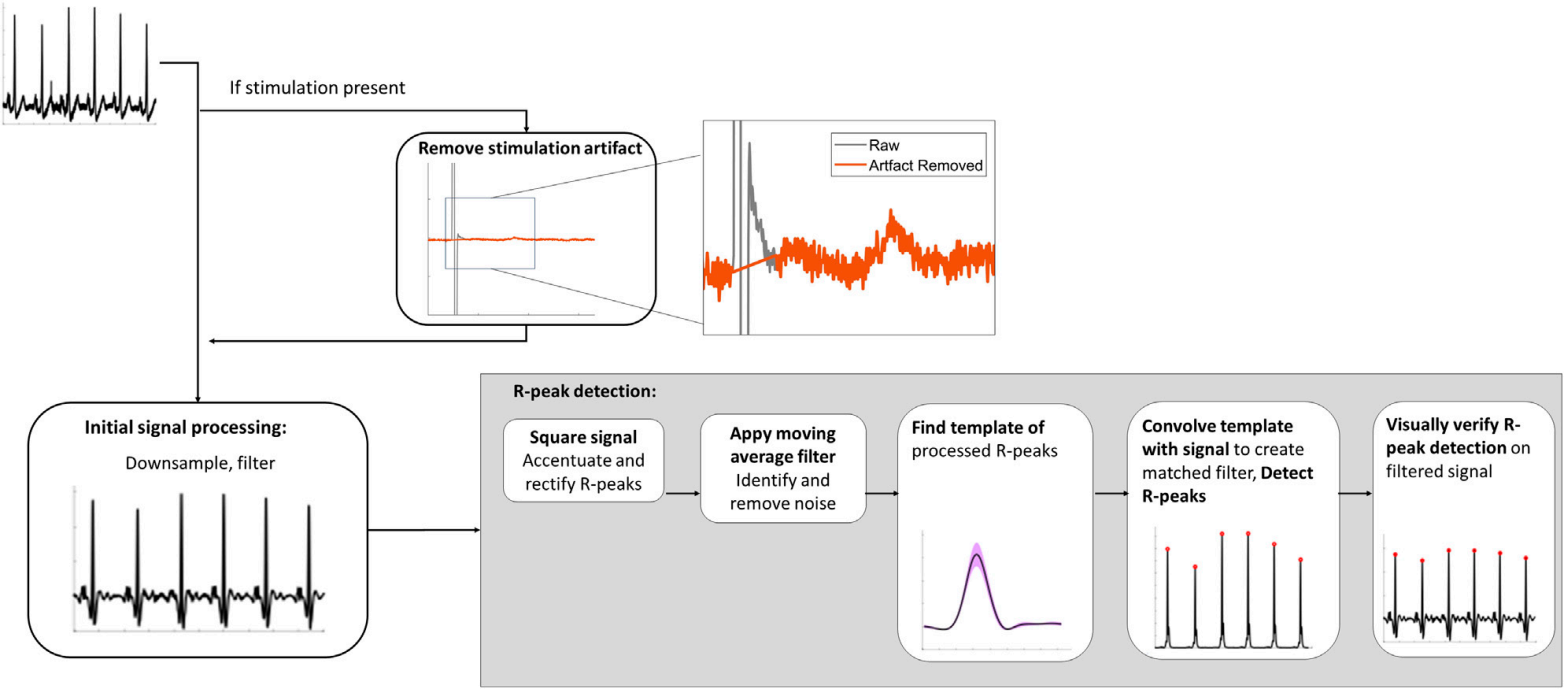
Spinal lead recording signal analysis processing pipeline to detect R-peaks.

Data analysis of the spinal recordings was conducted in MATLAB R2020b (MathWorks; Natick, MA, USA). R-peaks were identified as outlined in Figure 2 using a modified approach to the Pan-Tompkins method of QRS detection (Pan and Tompkins, 1985). For all recordings with stimulation, the stimulation artifacts were removed by linear interpolation. All signals were down-sampled to 1,000 Hz and filtered using a third order Butterworth bandpass filter from 5 to 50 Hz. Any potential remaining line noise at 60 Hz was removed with a third order Butterworth band-stop filter from 59 to 61 Hz. These filtered signals were squared to accentuate and rectify R-peaks for detection. Next, the *movmean* function was applied as moving average filter to identify and remove remaining noise, such as movement artifacts.

Following de-noising, R-peaks were isolated using a two-stage approach. First, the template of the processed R-peaks was found by using the *findpeaks* function with two constraints: the minimum IBI of 0.4 s between successive peaks (i.e., a maximum of 150 beats per min) and with an amplitude threshold of twice the RMS of the squared signal to ensure spurious signals or T-waves were not identified as peaks. The template was 82 ms to encompass the full R-peak which is 70 ms or less (Pérez-Riera et al., 2016). Secondly, the signal-derived R-peak template was convolved with the rectified signal to form a matched filter output. Finally, the R-peaks were detected from the matched filter using the *findpeaks* function with the following two constraints: a minimum distance of 0.4 s and an amplitude threshold of a moving average filter. The moving average filter was similar to the dynamic threshold approach used by Nguyen et al., with the moving average having a window size of 0.83 s (Nguyen et al., 2019). The amplitude threshold was found by scaling the moving average by 5.25 and adding to the baseline. Finally, R-peak detection was visually verified for each trace.

To quantify the signal quality, SNR was calculated with the signal estimated by the QRS template and noise estimated by removing the QRS template from the signal when R-peaks were detected. Given that the template was 82 ms, the estimated noise would incorporate any potential P- and T-waves present in the trace, which may over-estimate the noise but ensured sufficient time points were included. Previous studies found that ECG signals with SNR values above 5 had sufficient fidelity for QRS detection (Smital et al., 2020) and HRV analysis (Cavalieri and Filho, 2020). Visual inspection of our filtered data supported that SNR values above 5 enabled consistent detection of R-peaks. Thus, signals with SNR below 5 were defined as ‘poor quality’ and above 5 as ‘good quality’.

To identify the presence of any additional cardiac waves beyond the R-peak, an ensemble averaging approach was used to find mean segments that included the P-, QRS- and T-waves. Briefly, raw data was preprocessed similar to the R-peak detection but targeting lower frequencies: after removal of any stimulation artifact and down-sampling to 1,000 Hz, all signals were filtered using a third order Butterworth bandpass filter from 0.5 to 10 Hz (Elgendi et al., 2016). For each recording, traces were taken from 300 ms prior to 400 ms after each detected R-peak. Traces with a maximum deflection more than 15 ms from the R-peak were considered corrupted by noise and removed prior to averaging. The mean P-QRS-T-waves were plotted and visually inspected to determine if a P- and T- wave was readily apparent. SNR analysis for these waves was not performed as less than 10% of signal would remain to estimate noise for heart rates of 78 bpm or more.

Data are summarized by mean and standard deviation. For R-peak analysis of SNR, statistical significance for stimulation on vs. stimulation off, as well as neighboring configurations (e0e1 vs. e6e7) was tested using the Wilcoxon signed-rank test. Significant differences in SNR across body postures and electrode configurations was performed with Kruskal–Wallis tests, followed by Dunn’s *post hoc* testing.

### Analysis of wearable recordings

In a subset of 5 subjects, the performance of the spinal lead was compared to that of the Apple Watch (series 7, watchOS version 8.5), as a validated wearable for measuring HRV measurements (Hernando et al., 2018). Subjects wore the Apple Watch on their wrist and were asked to remain still throughout the duration of each approximately 5-min recording. Similar to Hernando et al. (2018), the Breathe session within the Mindfulness app was used to record beat-to-beat measurements of HR while cardiac signals were simultaneously recorded from the SCS lead. The Breathe app stores raw RR values within the session that would otherwise not be accessible. Similar to other wrist-worn wearables, the Apple Watch uses a photoplethysmography (PPG) signal to measure the pulses generated by blood flow in the wrist. Pulse-to-pulse (PP) intervals were found by taking the difference in time between successive beats detected by the Apple Watch.

**FIGURE 3:**
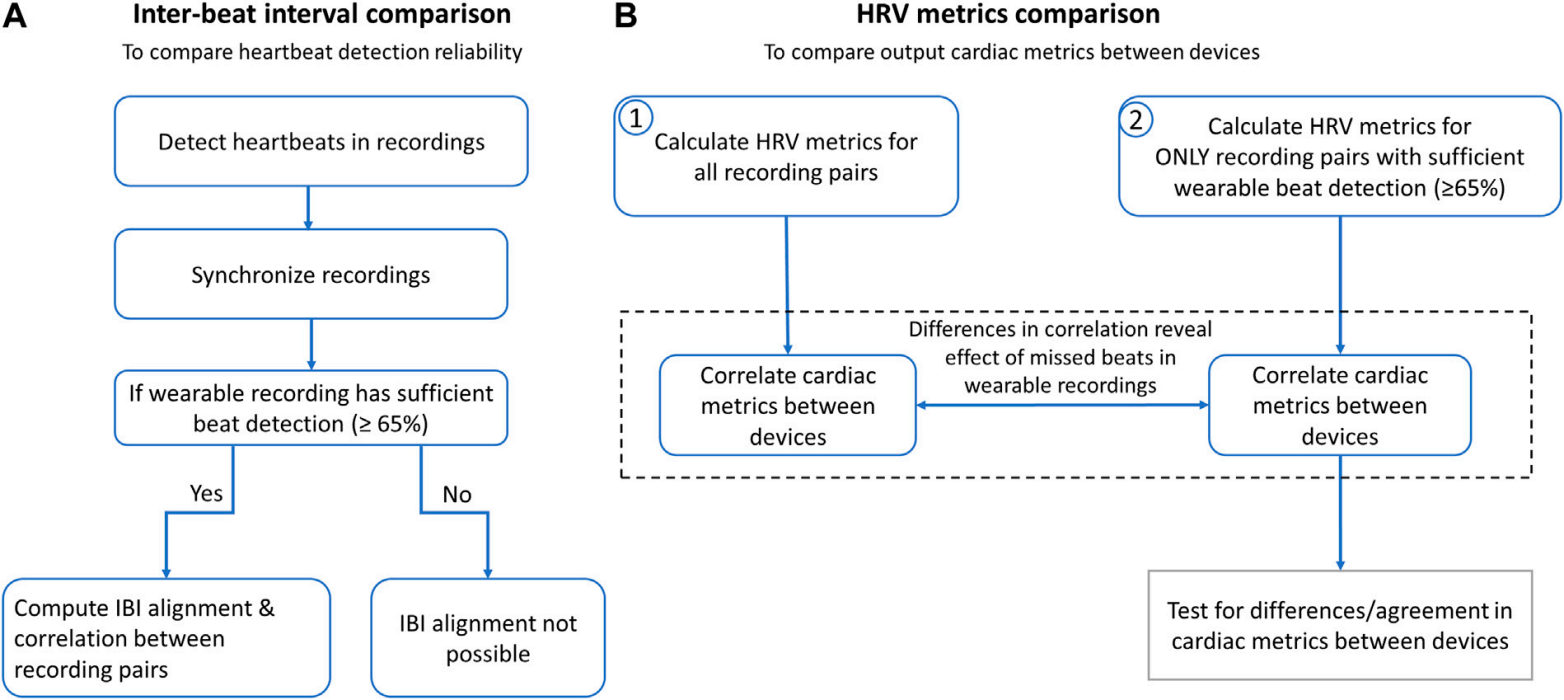
Conducted analyses to compare the spinal lead and wearable cardiac recordings and resulting cardiac metrics. **(A)** Inter-beat intervals (IBIs) were compared to determine reliability of heartbeat detection across devices. After heartbeats were detected, Apple Watch and SCS recordings were synchronized by aligning the first 15-20 IBIs and trimming ends to match durations. For recordings with sufficient beat detection, all IBIs from entire recordings were aligned and correlation statistics were calculated. **(B)** Heart rate (HR) and heart rate variability (HRV) metrics were calculated to determine correlation across devices with and without the wearable recordings with insufficient beat detection (defined as >35% missed beats).

For each ‘recording pair’, data from the Apple Watch and corresponding SCS recoding were synchronized using the first 15-20 IBIs for all but two recordings (wherein the Apple Watch had too many missed beats at recording onset) and trimmed to ensure the same recording duration between SCS and AppleWatch recordings. The analyses are summarized in Figure 3.

### Heartbeat detection and inter-beat interval comparison

The performance of the spinal recordings was validated by comparing the number of beats detected to those detected in the wearable recordings. Statistical significance was tested using the Wilcoxon signed-rank test.

Next, IBI correlation analysis (Pearson’s correlation), Bland-Altman plots (Ran Klein, 2023), and Intraclass Correlation Coefficient (ICC) (Arash Salarian, 2023) were calculated to compare IBI measurements across the two devices. For these analyses, the IBIs between the wearable and the spinal recordings were aligned in time similar to other IBI alignment approaches (Hernando et al., 2018). Specifically, gaps were introduced to segments where the corresponding recording had ectopic beats. Ectopic beats were identified via outlier RR-intervals, defined by having an IBI more than 0.3 s above or below the mean IBI (e.g., a typical IBIs of 0.9 s would remove values outside 0.6–1.2 s) for each 5-min recording. All alignment was visually inspected, and recordings with greater than 35% missed beats were excluded from IBI correlation analysis due to difficulty of visual alignment. ICC values above 0.7 are considered reproducible, while values above 0.8 are considered good and above 0.9 are considered excellent (Sandercock et al., 2005; Yperzeele et al., 2016; Goudman et al., 2019).

### Cardiac metrics comparison: heart rate variability metrics

Differences in time and frequency domain parameters of HRV were compared from all synchronized spinal lead and wearable recording pairs. A variety of HRV metrics (see Shaffer and Ginsberg, 2017 for a review) can be derived - here a number of commonly-used time and frequency domain HRV metrics were calculated.

HR was calculated from the wearable by averaging beat-to-beat HR over the course of the recording. In addition, the following time domain HRV metrics were calculated: the average RR interval (AVRR), the standard deviation of the RR intervals (SDRR), and the root mean square of successive differences (RMSSD). For time domain metrics, ectopic beats were identified via outlier RR-intervals and removed from analysis after visual inspection. Similar to IBI alignment, outlier RR-intervals were defined by having an IBI more than 0.3 s above or below the mean IBI.

The following frequency domain HRV metrics were derived: absolute high frequency (HF_abs_) and low frequency (LF_abs_) values, along with normalized values (HF_nu_ and LF_nu_). For frequency domain metrics, outlier IBIs were identified and replaced via linear interpolation to minimize the effect on metrics. HRV Analysis Software (HRVAS) (Ramshur, 2010) was then used to further process and analyze the signals. Similar to (Goudman et al., 2019), ectopic beats were identified with a median filter and corrected with cubic spine interpolation. Signals were then detrended, linearly interpolated and resampled at 2 Hz. Spectral power was calculated using the Burg method between 0.04 and 0.15 Hz for low frequencies and between 0.15 and 0.4 Hz for high frequencies.

To compare the wearable and spinal lead derived values, Pearson correlation statistics for each HRV metric were calculated, with *p*-values corrected for multiple comparisons using Bonferroni’s correction. A linear regression was fitted, where the explanatory variable (x) was the spinal-lead derived metric and the dependent variable (y) was the wearable derived metric.

The effect of the missed beats in the wearable recordings on these cardiac metrics were further investigated by excluding recordings with greater than 35% missed beats and re-calculating HRV metrics. Pearson correlation tests were again performed and linear regression was also fitted with this subset of recordings.

Finally, Bland-Altman plots were created to compare agreement in HRV metrics between aligned-only recordings, with the bias and Limits of Agreement (LOA) summarized for each data set. The ICC was also computed for these HRV metrics. Differences in metrics across devices were determined using the Wilcoxon signed-rank test. For both difference testing and ICC, *p*-values were corrected for multiple comparisons using Bonferroni’s correction.

## Results

Cardiac signals were recorded from SCS leads in 10 chronic pain subjects. The average age of the 10 subjects was 71.4 ± 10.8 years (range: 56.3–87.8 years) with 8 females and 2 males. The primary indication was Failed Back Surgery Syndrome for 5 patients, post-laminectomy pain for 4 subject, and Multiple Back Operations for 1 subject. The effect of body posture and recording electrode configuration on the signal quality was then assessed. In a subset of 5 subjects, the number of beats detects, IBIs, and HRV metrics derived from the spinal recordings were compared to those from the wearable recordings.

### Cardiac signals across a variety of postures and electrode configurations

A total of 157 recordings of at least 30 s in duration (average 149 ± 120 s, range 31–404 s) were taken of cardiac signals in various positions and electrode configurations. Of these recordings, 14 had concurrent stimulation while the other 143 were recorded in the absence of stimulation. All 14 recordings with electrical stimulation had SNR above 5 (average 14 ± 3) and were not significantly different than recordings with stimulation off (*p* > 0.05, Wilcoxon rank sum test). Hence, all of these recordings were included in subsequent analyses. In addition, of the 53 recordings performed on neighboring electrode recordings in resting positions, 43 recordings were acquired on cranial contacts (i.e., e0e1), while 10 recordings were performed on caudal electrodes (i.e., e6e7). The signal quality was not significantly different (*p* > 0.05, Wilcoxon rank sum test) between these two orientations, and thus were also grouped together as ‘neighboring’ in subsequent analyses.

**FIGURE 4:**
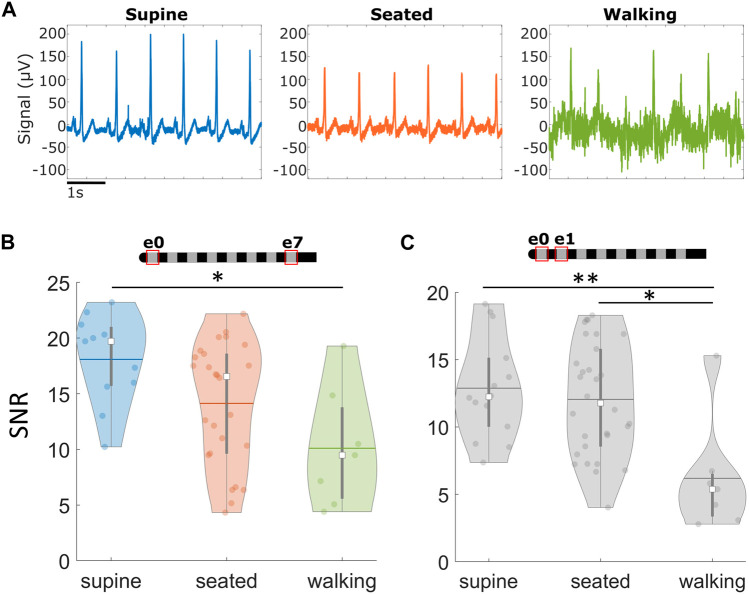
Cardiac signals recorded via SCS leads across different postures. Signals at rest are more robust than during movement. **(A)** Examples of raw signals recorded across different positions in the e0e7 recording configuration, in the absence of stimulation. **(B)** Summary of average Signal-to-Noise Ratio (SNR) across different postures using the across-lead recording configuration and **(C)** neighboring contacts. *p < 0.05,**p < 0.01. Note: Within each violin plot, the center white square is the median with the thick vertical gray line representing the interquartile range (similar to a boxplot) and the horizontal line is the mean. The surrounding colored dots are values from individual recordings.

When comparing with the same electrode configuration, the R-peaks were comparable in amplitude for all three positions (i.e., seated, supine, and walking) (e.g., raw examples recorded in an across-lead configuration as shown in Figure 4A). However, the signal quality (i.e., SNR) was significantly higher for supine recordings compared to walking for both across-lead (e0–e7; *p* = 0.01) and neighboring (e.g., e0–e1; *p* < 0.01) sensing configurations (Figure 4B). The seated position was also significantly higher in SNR compared to walking for the neighboring sensing configuration (*p* = 0.01) (Figure 4C). No significant differences were observed between supine and seated for either neighboring or across-lead configurations.

**FIGURE 5:**
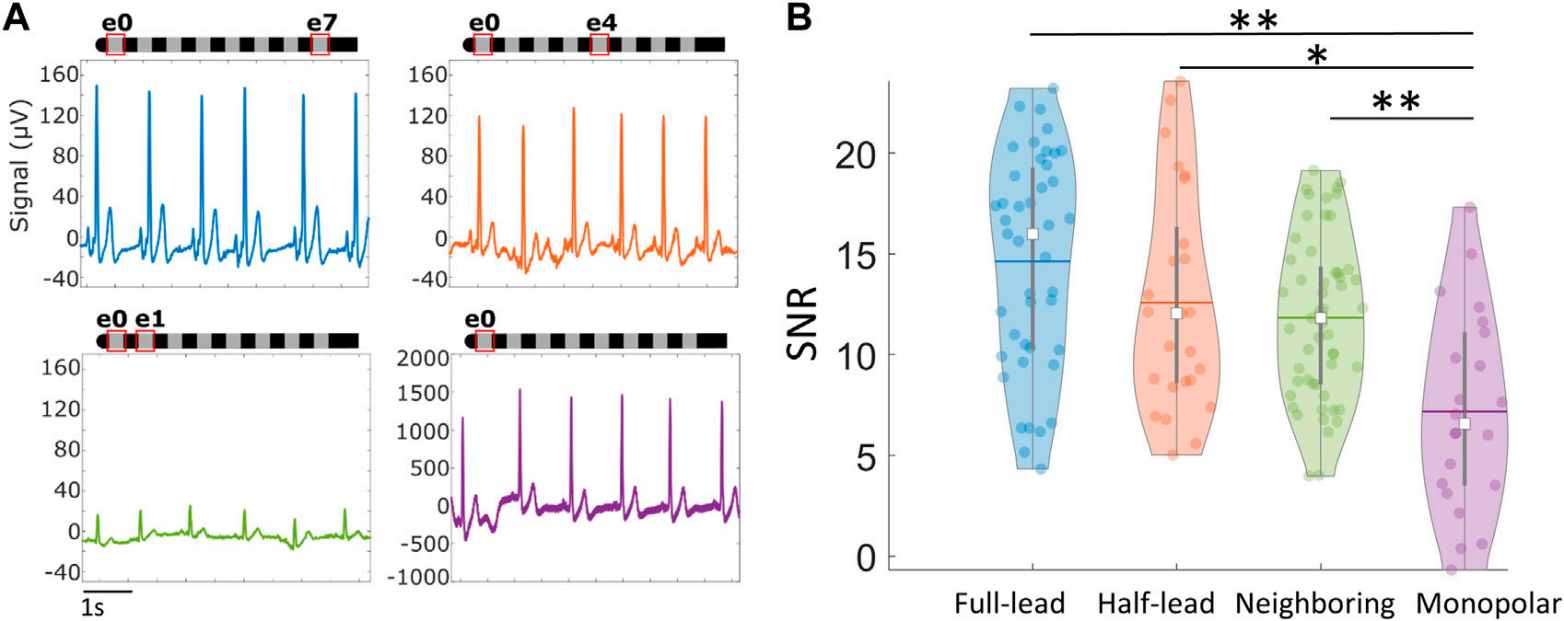
Cardiac signals recorded via SCS leads across different recording electrode configurations. **(A)** Examples of raw signals recorded using different electrode configurations in the absence of stimulation. **(B)** Violin plots summarizing average Signal-to-Noise Ratio (SNR) across different recording configurations while subject is stationary. *p < 0.05, **p < 0.01. Note: Within each violin plot, the center white square is the median with the thick vertical gray line representing the interquartile range (similar to a boxplot) and the horizontal line is the mean. The surrounding colored dots are values from individual recordings.

To identify those configurations that produce high quality signals with the least impact by noise, signals were compared across four different electrode configurations: across the full lead (e0–e7), half the lead (e.g., e0–e4), neighboring electrodes (e.g., e0–e1), and monopolar (e.g., e0–patch electrode). The electrode recording configurations and examples of the raw signals recorded can be found in Figure 5. Sensing across the lead (e0–e7) yielded large amplitude cardiac signals that were minimally susceptible to noise. While the monopolar configuration resulted in a larger signal, it was easily corrupted by movement artifacts and other noise sources that led to significantly reduced SNR compared to other sensing configurations (*p* < 0.05). Despite resulting in the smallest cardiac signal, neighboring electrode pairs (e.g., e0–e1) still maintained an acceptable SNR (>5) for successful R-peak detection for 51 of 53 recordings, with an average SNR of 12 ± 4. Moreover, there was no significant difference in signal quality in neighboring configurations compared to the full-lead or half-lead configurations (*p* > 0.05).

### Detection of additional waves in the cardiac signals

**FIGURE 6:**
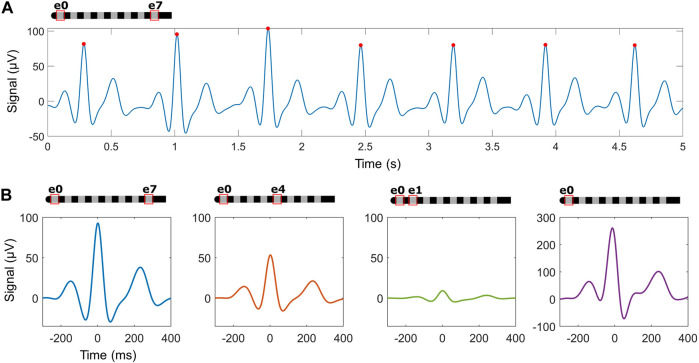
Ensemble averaging using detected R-peaks may identify additional waves within the heartbeat across different recording electrode configurations. **(A)** An example trace in the cross-lead configuration after processing for P- and T-wave detection, with detected R-peaks (red circles). **(B)** Examples of ensemble averaged waveforms across configurations in one subject.

Across the cardiac recordings, additional features of the heartbeat beyond R-peaks were evident including the presence of P-waves and T-waves. To best identify them, raw data was pre-processed at lower frequencies (see Methods for details) as seen in Figure 6A. The waveforms for each beat were then aligned using the pre-identified R-peak and ensemble averaged for each recording as seen in Figure 6B. Across 143 recordings in 10 subjects while in resting positions, 64% (n = 92 recordings across 9 subjects) had visually-evident P-waves and T-waves. For these recordings, the size of the ensemble-averaged P-wave was on average 18% ± 12% of the R-wave, and the T-wave was 37% ± 17%.

### Association and agreement of cardiac signals between wearable and spinal lead

To compare heartbeat detection across devices, 32 simultaneous recordings were collected from the wearable and the spinal lead across a subset of 5 subjects. Three recordings from a single subject had to be removed from analysis due to excessive movement, leaving a total of 29 recording pairs. These recording pairs were synchronized by aligning the first 15-20 IBIs (see Methods for details) and trimming the durations to match, with an average duration of 282 ± 3 s.

**FIGURE 7:**
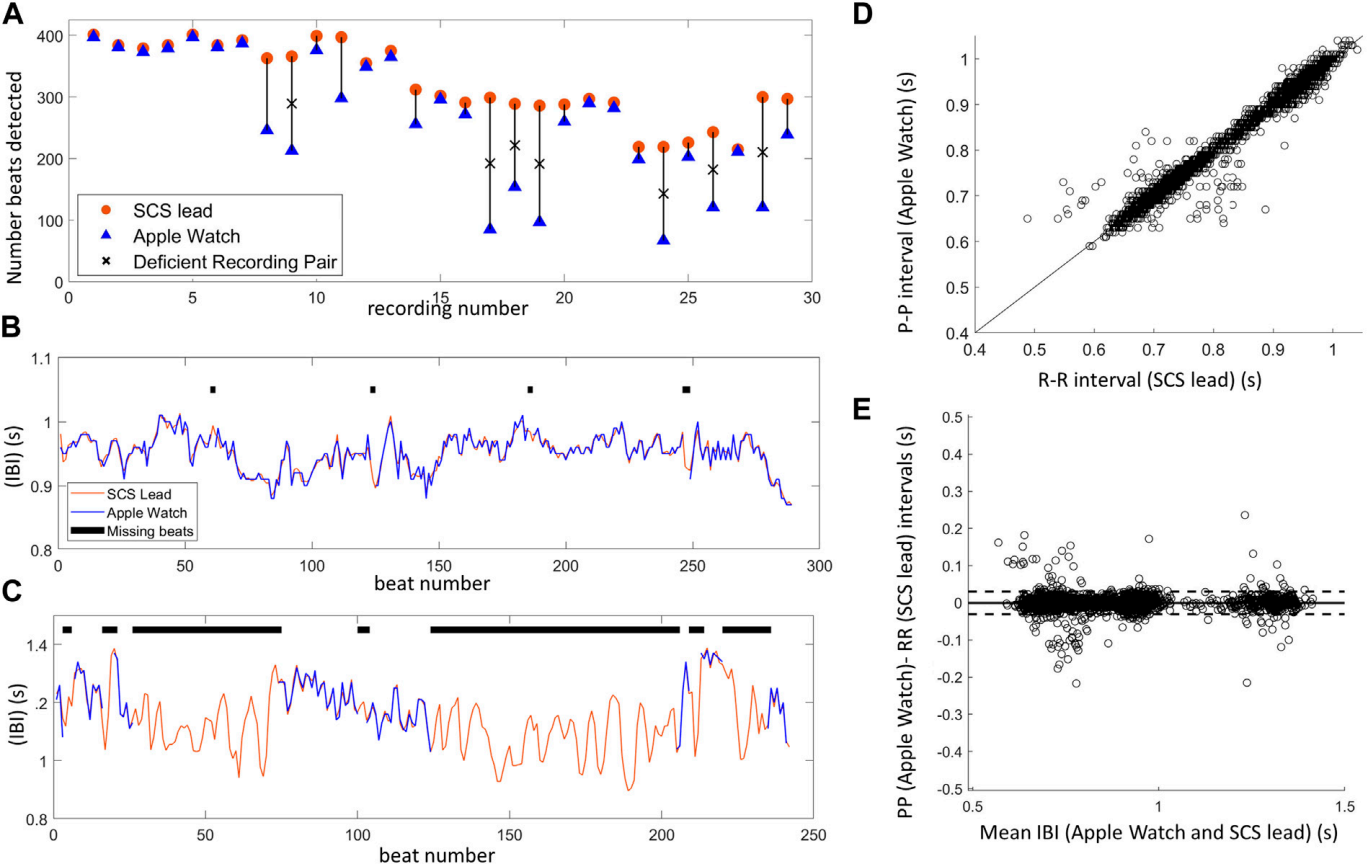
Inter-beat interval (IBI) comparison between SCS lead system and Apple Watch highlights one limitation of IBI measurements using PPG–missed beats. **(A)** The number of beats detected for each Apple Watch (blue) and SCS lead (orange) recording pair shows several Apple Watch recordings with insufficient beat detection denoted by an ‘X’ (missed >35% of beats). **(B)** Example of aligned IBIs over a 5 min recording when Apple Watch detected a sufficient number of beats (missed 2.4%). **(C)** Example of aligned IBIs when Apple Watch performed poorly, missing a majority of beats (50.2%) during the 5 min recording. This recording was not included in IBI correlation analysis and only segments with at least 3 consecutive IBIs were included in this manual alignment to demonstrate the difficulty in aligning data with missed beats. **(D)** There was high correlation of IBIs between the Apple Watch (PP intervals) and SCS lead (RR intervals) synchronized, aligned data (22 recordings). Line shown is x = y. **(E)** Bland-Altman plot showing agreement between the Apple Watch and SCS lead recordings, with the horizontal line showing mean difference (i.e., bias), and the dashed lines representing 95% limits of agreement.

The number of detected beats was significantly lower (Wilcoxon signed-rank test: *p* < 0.001) when using the wearable (avg: 265 ± 104; range: 67–397 beats) compared to the spinal lead (avg: 323 ± 62 beats; range: 215–401) (Figure 7A). In 22 of those recordings, the wearable detected a sufficient number of beats to enable visual IBI alignment. Specifically, the wearable recording missed an average of 7.2% ± 8.8% beats (range 0.8%–32.2%) compared to the corresponding SCS recording. The remaining seven wearable recordings missed over 35% of beats (avg: 58.0% ± 11.8%; range: 41.8%–71.5%) compared to the spinal lead recordings, making it difficult to reliably align the IBIs (examples of alignment shown in Figures 7B, C). Therefore, these recordings were not included in the subsequent IBI correlation analysis or Bland-Altman plots (Figures 7D, E). Of the 29 recording pairs, 5 recordings had concurrent electrical stimulation: two of these recordings allowed for IBI alignment while three did not.

For the 22 recordings with sufficient beats, visual IBI alignment was performed and outlier IBIs in the wearable data were detected (n = 202) and then removed from the recording pair. In the corresponding spinal lead recordings, 10 outlier IBIs were detected across four recordings. Some of these corresponded to outlier IBIs detected on the wearable recording, and thus 4 outlier IBIs across two recordings were additionally removed. Once the outliers were accounted for in both the wearable and spinal lead recordings, IBIs were highly correlated (Pearson correlation: R = 0.99, *p* < 0.001, n = 6609; Figure 7D). Strong agreement between the two device recordings can further be observed with the Bland-Altman plot (Figure 7E) (bias = 0.0, LOA -0.03 to 0.03), as well as the ICC of 0.996 (95% CI of 0.9958–0.9962).

### Heart rate variability metrics

**FIGURE 8:**
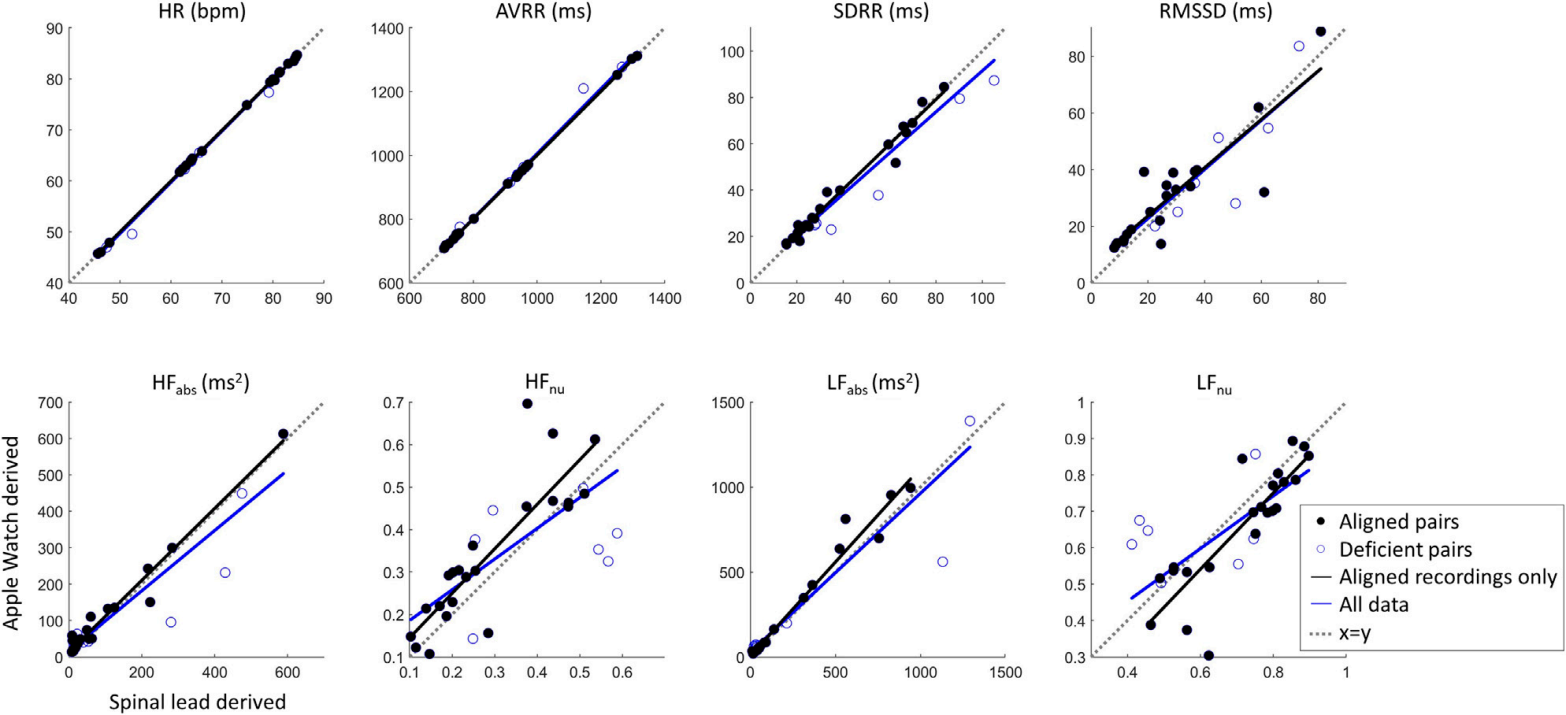
Cardiac metric correlation between Apple Watch and SCS lead system show that while time domain metrics are not likely affected by missed beats, the frequency domain metrics may be altered by this limitation. Dashed line of x = y indicates parity.

To evaluate whether differences in beat detection would affect summary measures, HRV metrics in the time and frequency domains were evaluated across the synchronized wearable and spinal lead recordings. As detailed above, all 29 recording pairs were initially included in this analysis, and then a second analysis was done for only the 22 recording pairs with IBIs where visual alignment was possible (Figure 8).

Correlation statistics summarizing Pearson correlation with Bonferroni’s correction for multiple comparisons can be found in Table 1. The spinal lead and wearable were significantly correlated for all cardiac metrics tested, whether including or excluding recording pairs with insufficient beats detected by the wearable. When all 29 recording pairs were included, time-domain and absolute frequency-domain HRV metrics showed strong correlation (R > 0.89) between wearable and spinal lead derived values; normalized HF and LF revealed weaker correlation across devices (R = 0.71). When evaluating cardiac metric correlations for only the 22 recordings pairs with sufficient wearable beat detection, correlation coefficients were at or above 0.86 for all cardiac metrics reported. Notably, R values for all frequency domain metrics rose when excluding deficient recording pairs, with the greatest increase in correlation occurred for HF_nu_ and LF_nu_ where R grew from 0.71 to 0.86 after excluding insufficient beat recordings by the wearable.

**TABLE 1:** Cardiac metrics across devices–Correlation Statistics.

	All recording pairs		IBI aligned pairs only	
	Pearson correlation	Fit equation	Pearson correlation	Fit equation
HR	R = 1.00, *p* < 0.001	y = 1.01 x–0.6	R = 1.00, *p* < 0.001	y = 1.00 x + 0.17
AVRR	R = 1.00, *p* < 0.001	y = 1.02 x–11.44	R = 1.00, *p* < 0.001	y = 1.00 x + 0.10
SDRR	R = 0.97, *p* < 0.001	y = 0.89 x + 2.8	R = 0.99, *p* < 0.001	y = 0.97 x + 1.76
RMSSD	R = 0.89, *p* < 0.001	y = 0.87 x + 5.32	R = 0.89, *p* < 0.001	y = 0.85 x + 6.52
HF_abs_	R = 0.93, *p* < 0.001	y = 0.83 x + 15.78	R = 0.98, *p* < 0.001	y = 0.99 x + 12.62
HF_nu_	R = 0.71, *p* < 0.001	y = 0.72 x + 0.11	R = 0.86, *p* < 0.001	y = 1.05 x + 0.04
LF_abs_	R = 0.94, *p* < 0.001	y = 0.94 x + 27.41	R = 0.99, *p* < 0.001	y = 1.11 x + 4.63
LF_nu_	R = 0.71, *p* < 0.001	y = 0.72 x + 0.16	R = 0.86, *p* < 0.001	y = 1.05 x–0.09

**FIGURE 9:**
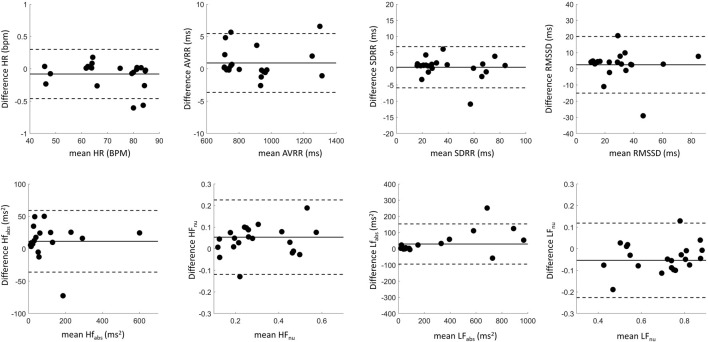
Bland-Altman plots showing agreement between cardiac metrics recorded by the SCS lead and the Apple Watch. Data in black summarizes aligned pairs. The solid lines represent the difference in cardiac metrics between the recording modalities (bias), while the dotted lines represent limits of agreement.

For the 22 recordings with sufficient beats, agreement in HRV metrics between the devices was further investigated with Bland-Altman plots in Figure 9, with summary statistics in Table 2, demonstrating a high overlap in agreement between the two devices. Also summarized in Table 2, the ICC also shows good or excelled correlation (above 0.8) for all time domain metrics as well as for HF_abs_ and LF_abs_, with HF_nu_ and LF_nu_ just below that cutoff at 0.79 but still considered reproducible. Finally, we tested whether HRV metrics were statistically different across devices (Table 2). All frequency-domain metrics significantly differed across devices (*p* < 0.05, Paired Wilcoxon test with Bonferroni’s correction); however, differences in time domain metrics between devices were not statistically significant. Thus, while there was high correlation and agreement between devices for HRV metrics, the exact values for frequency metrics were statistically different.

**TABLE 2:** Intraclass coefficients, Bias, and Wilcoxon test *p*-values (after Bonferroni’s correction) on HRV Parameters derived from aligned SCS leads and Apple Watch recordings with sufficient beat detection (n = 22). LOA, Limits of Agreement; CI, Confidence Interval.

	Wilcoxon test *p*-value	Bias (LOA)	ICC (95% CI)
HR	*p* > 0.05	−0.1 (−0.5 to 0.3)	0.9999 (0.9996–1)
AVRR	*p* > 0.05	0.9 (−3.6–5.5)	0.9999 (0.9997–1)
SDRR	*p* > 0.05	0.5 (−5.9–6.9)	0.99 (0.97–0.997)
RMSSD	*p* > 0.05	2.5 (−15.0–20.0)	0.88 (0.65–0.96)
HF_abs_	*p* = 0.03	11.5 (−36.1–59.2)	0.98 (0.94–0.99)
HF_nu_	*p* = 0.04	0.1 (−0.1–0.2)	0.79 (0.44–0.93)
LF_abs_	*p* = 0.02	29.3 (−94.8–153.5)	0.98 (0.92–0.99)
LF_nu_	*p* = 0.03	−0.1 (−0.2 to 0.1)	0.79 (0.44–0.93)

## Discussion

In this study, cardiac signals recorded via SCS leads implanted in the thoracic spine were characterized to determine the feasibility of chronic cardiac monitoring using a fully implanted SCS system in future applications. Our results showed that SCS leads, as a part of a recording system, are capable of sensing cardiac signals wherein detected R-peaks can be processed for HR and HRV metrics. Moreover, the lead-derived signals out-performed wearable beat detection in this feasibility study, which could enable long-term monitoring at home and during activities of daily living. The SCS leads were also capable of detecting other features of the cardiac signal, including P-waves and T-waves, for a majority of recordings based on visual assessment.

### Quality of cardiac signals recorded from spinal leads

This study demonstrated that SCS lead recordings in resting positions, especially supine, resulted in higher signal quality compared to walking as quantified with SNR. This finding is similar to results using a traditional 12-lead ECG (Fouassier et al., 2020; Hamada et al., 2022). Distance between electrode pairs influenced signal amplitude, since the across-lead recording pairs (e.g., e0–e7) resulted in larger amplitude signals compared to neighboring pairs (e.g., e0–e1). This is expected and seen in other studies using traditional ECG devices (Nedios et al., 2014; Hamada et al., 2022) because larger inter-electrode spacing affords a wider electrical vector to acquire the electrical dipole generated by the heart.

Notably, this increase in amplitude did not result in a statistically significant effect on SNR, suggesting that these changes were accompanied by similar increases in noise. Moreover, 51 of 53 of the smaller amplitude signals observed in narrow electrode pair recordings still resulted in an acceptable signal quality (SNR >5) when the subject was stationary. Overall, all electrode configurations during rest produced recordings with an average SNR greater than 5, which is acceptable for R-peaks to be easily distinguished from noise. This flexibility in recording configuration is critical in future applications with therapeutic stimulation, given that proximity to stimulation contacts increases the probability for artifacts. For example, recording cardiac signals with narrowly-spaced electrodes on one end of the spinal lead may allow for stimulation on the opposite end, as is currently done with closed-loop ECAP-responsive SCS therapy (Russo et al., 2018; Vallejo et al., 2021). It was also observed that walking significantly decreased SNR, likely due to added noise from movement artifacts. Thus, future implementations of on-device cardiac sensing should incorporate robust noise detection algorithms to ensure errant signals are not incorrectly labeled as heart beats.

### Utility of cardiac metrics in quantifying chronic pain and wellbeing

Many studies have investigated the complex nature between HRV metrics and pain towards the goal of defining more objective measures of pain. Several seminal studies have demonstrated that parasympathetic related HRV metrics decrease with experimentally induced acute pain (Jiang et al., 2017; Luo et al., 2020; Forte et al., 2022). However, the translation of these results to fluctuations of pain within the chronic pain population is unclear, especially since this population has an increased likelihood of autonomic nervous system imbalance as evidenced by lower baseline parasympathetic related HRV metrics (Hallman et al., 2015; Koenig et al., 2016; Tracy et al., 2016; Karri et al., 2017). A number of studies investigated how HRV fluctuates in response to changes in chronic pain with SCS therapy with inconsistent trends. Goudman et al. found that SCS increased the HF HRV, while lowering LF HRV, and not affecting LF/HF ratio across 22 subjects. An increase in HF HRV aligns with the theory that pain treatment, whether SCS or otherwise, helps to restore parasympathetic activity and hence restore the imbalance between both output mechanisms of the autonomic nervous system. Conversely, Kalmar et al. (2013) found that the SCS decreased HF HRV, and did not affect other HRV parameters, while Black et al. (2022) found no significant differences in HRV metrics with SCS therapy. Both of these studies were limited by small sample size with only 6-7 subjects completing follow-up. Meanwhile, Moens et al. (2023) found no correlation between HRV recordings of over 350 subjects and their pain intensity over 7 days. However, only one 5 min recording was taken in this study, and thus results did not capture any fluctuations in pain or HRV that subjects may have over time. Notably, these studies investigated HRV in the clinical setting with a so-called “snapshot recording”, where results may be confounded by the additional stress compared to a more comfortable home setting. Recently, Patterson et al. (2023) used wearables to track chronic pain patients implanted with a SCS device over 6 months and found that HR and HRV (SDRR specifically), among other objective measures, was identified as a contributing feature in modeling pain levels. Here, we demonstrate that this type of information, as well as the raw metrics used to compute a variety of physiological metrics, can be collected without the need for a wearable. On the long-term, this would allow continuous patient monitoring (i.e., ecological momentary assessment), without the need for wearables, to gain better insights in the impact of chronic pain in patients treated with SCS. Future studies should further explore how and to what extent the array of HRV parameters change with chronic pain over a duration of months or years for chronic pain patients with SCS devices.

While wearables are gaining traction in pain assessment (see Leroux et al., 2021 for review) and provide the ability to do longer at-home studies with subjects to establish a baseline HRV measurement, these external devices have key limitations compared to an implantable SCS system: patient compliance, manufacturer-specific calculations and timing of measurements, and reliability of beat detection. First, patient compliance in wearing devices may be low, such as in the Han study where chronic pain patients only wore the wearable for an average of 143 of 365 days (Han et al., 2022). A SCS system with integrated sensors and metrics would reduce patient burden and thus capture more data. Secondly, while most smartwatches report out the RMSSD metric of HRV, the timing of measurements and calculations differ and access to raw data is limited. Thirdly, despite the Apple Watch being validated previously for HRV measurements against traditional ECG, we see in our study and others (Hernando et al., 2018; Cajal et al., 2022; Patterson et al., 2023) that the Apple watch has limitations when it comes to reliable detection of heart beats. The reliability of beat detection may greatly impact cardiac metrics, given that errors representing <0.1% of IBIs may impact HRV parameters dramatically (Kemper et al., 2007). To address this, a variety of correction methods may be used, including removing outliers or using gap filling methods (Cajal et al., 2022). Even with these methods, limiting the loss of missed beats is important especially as some metrics are less resilient than others. Specifically, Cajal et al. showed that while the mean heart rate can tolerate up to 35% missed beats and have a relative error of less than 20% with corrections applied, frequency domain metrics such as HF_nu_ can tolerate only up to 15% errors even with advanced gap filling approaches.

Despite these limitations, wearables will continue to be a valuable tool for studies investigating objective measures of chronic pain, and indeed we observed minimal effects in time domain metrics of missed beats in the Apple Watch data. Caution should be exercised for frequency domain analysis of HRV parameters, as missed beats caused decreased correlation with the spinal lead as well as significant differences in HRV metrics for IBI-aligned recordings. Time domain and frequency domain analysis give several parameters corresponding to different aspects of autonomic balance (Stein and Pu, 2012; Koenig et al., 2016; Shaffer and Ginsberg, 2017; Hohenschurz-Schmidt, 2020), and accessing these rich metrics may provide clarity in future research studying the relationship between HRV and chronic pain. Moreover, these metrics could provide insights into generalized health beyond pain, as HR and HRV are useful in assessing sleep quality (Tobaldini et al., 2013; Mitsukura et al., 2020) and are indicators of heart health and risk level for cardiovascular diseases (Dekker et al., 2000; Kleiger et al., 2005). Having reliable, on-device recordings could also further research into objective measures of stress, which is not recommended using traditional statistical methods with an Apple Watch in a healthy population (Velmovitsky et al., 2023). Furthermore, future studies could assess the full cardiac waveform from implanted SCS lead recordings towards arrythmia detection considering chronic pain patients are at a higher risk of cardiovascular disease (Rönnegård et al., 2022).

### Limitations

While this study focused on the feasibility of collecting cardiac data in a small number of subjects, there are some limitations to consider. First, only subjects with thoracic-implanted leads were included, and thus the ability to record these signals in cervically-implanted subjects is unclear. Moreover, signals were only recorded on externalized leads at the end of the trial, and fully-implantable systems should be evaluated with a gold-standard ECG across a larger sample size. Additionally, the statistical tests used here intrinsically assume independence across recordings, which may affect some confidence intervals and thus significance testing. In this feasibility work, we did not sufficiently power the study to characterize differences between lead positioning or to thoroughly determine the effect of stimulation on *versus* off, though the data presented here suggests any potential differences would not cause the signal to degrade in quality to such a degree that would make R-peak detection unreliable (i.e., an SNR below 5). In addition to detecting R-peaks, we also observed smaller features including P-waves and T-waves in 64% of recordings. The prevalence of these features likely varied with lead position, as the recording vector is non-ideal for P-waves or T-waves, as well as the quality of the signal as we anecdotally observed that recordings with large SNR for R-peaks often had these waves apparent. Thus, further analysis of these features would require concurrent ECG recordings for further validation. This study is an important step towards future research that should investigate changes in cardiac signal quality over time in a chronically implanted system with the presence of SCS therapy. In initial feasibility studies in sheep (n = 5), we have found cardiac signals could be recorded months after implantation of SCS leads (data not shown), though this should be further explored in humans. Moreover, future research should also further correlate summary metrics of these signals with pain scores to identify potential biomarkers.

## Conclusion

Cardiac metrics discussed herein could inform researchers and clinicians through automated feedback about their patients’ health and wellbeing. Combined with on-device accelerometry, a plethora of health measures can be collected, including heart, sleep, and activity metrics. In addition, these insights could also inform machine learning algorithms that attempt to predict patient outcomes and therapy adjustments to further improve SCS therapies. Moreover, these holistic measures could be used in a closed-loop device that automatically adjusts and improves long term durability of therapy to increase patient satisfaction. To achieve this goal, the data must be reliable to determine whether it is reflective of the patient’s response to therapy, either as a correlate to the person’s chronic pain or other wellbeing factors. With sensing capabilities built into existing implantable SCS systems, longitudinal cardiac monitoring may help us to investigate the relationship between cardiac metrics, pain, and wellbeing.

## Data Availability

The datasets presented in this article are not readily available because the datasets are the property of Medtronic plc. Requests to access the datasets should be directed to margo.m.straka@medtronic.com.
